# Expression of Concern: Suppression of microRNA-135b-5p protects against myocardial ischemia/reperfusion injury by activating JAK2/STAT3 signaling pathway in mice during sevoflurane anesthesia

**DOI:** 10.1042/BSR-20170186_EOC

**Published:** 2021-03-26

**Keywords:** Inhalation anesthesia, JAK2/STAT3 signaling pathway, MicroRNA-135b-5p, Myocardial ischemia/reperfusion injury, Sevoflurane

The Editorial Office has been made aware of potential issues surrounding the scientific validity of this paper, hence has issued an expression of concern to notify readers whilst the Editorial Office investigates.

